# *De novo* actin polymerization is required for model Hirano body formation in *Dictyostelium*

**DOI:** 10.1242/bio.014944

**Published:** 2016-05-23

**Authors:** Yun Dong, Sonbol Shahid-Salles, Dan Sherling, Nathan Fechheimer, Nathan Iyer, Lance Wells, Marcus Fechheimer, Ruth Furukawa

**Affiliations:** 1 Department of Cellular Biology, University of Georgia, Athens, GA, USA 30602; 2 Complex Carbohydrate Research Center, Department of Biochemistry and Molecular Biology, University of Georgia, Athens, GAUSA 30602

**Keywords:** *Dictyostelium*, Hirano body, Actin binding protein, Mass spectrometry, Arp2/3, Profilin, WASH, HSPC300

## Abstract

Hirano bodies are eosinophilic, actin-rich inclusions found in autopsied brains in numerous neurodegenerative diseases. The mechanism of Hirano body formation is unknown. Mass spectrometry analysis was performed to identify proteins from partially purified model Hirano bodies from *Dictyostelium*. This analysis identified proteins primarily belonging to ribosomes, proteasomes, mitochondria and cytoskeleton. Profilin, Arp/2/3 and WASH identified by mass spectrometry were found to colocalise with model Hirano bodies. Due to their roles in actin regulation, we selected these proteins for further investigation. Inhibition of the Arp2/3 complex by CK666 prevented formation of model Hirano bodies. Since Arp2/3 activation occurs via the WASH or WAVE complex, we next investigated how these proteins affect Hirano body formation. Whereas model Hirano bodies could form in WASH-deficient cells, they failed to form in cells lacking HSPC300, a member of the WAVE complex. We identified other proteins required for Hirano body formation that include profilin and VASP, an actin nucleation factor. In the case of VASP, both its G- and F-actin binding domains were required for model Hirano body formation. Collectively, our results indicate that *de novo* actin polymerization is required to form model Hirano bodies.

## Abstract (precis)

**Summary:** Hirano bodies are F-actin-rich inclusions found in a wide variety of neurodegenerative diseases with a complex physiological role. The composition and formation of Hirano bodies were investigated.

## INTRODUCTION

Neurodegenerative diseases affect millions of people worldwide and are caused by loss of structure or function of neurons. Some of the most widespread diseases exhibit hallmark protein aggregates such as Alzheimer's disease (AD) with β-amyloid plaques (Glenner and Wong, 1984) and neurofibrillary tau tangles (Kosik et al., 1986), Parkinson's disease with Lewy bodies containing α-synuclein (Spillantini et al., 1998), and amyotrophic lateral sclerosis (ALS) with the aggregation of TDP43 (Neumann et al., 2006). Actin inclusions are also present as pathological structures in the form of Hirano bodies (Galloway et al., 1987a; Goldman, 1983; Hirano et al., 1968) and actin-cofilin rods (reviewed in Bamburg et al., 2010). Hirano bodies are eosinophilic cytoplasmic inclusions that are composed primarily of F-actin, with a characteristic electron dense paracrystalline ultrastructure (Goldman, 1983; Schochet and McCormick, 1972). Hirano bodies colocalize with actin-binding proteins as well as proteins that are significant for neurodegenerative diseases, such as tau in AD (Galloway et al., 1987a,b); however since investigation of Hirano bodies was limited to postmortem tissues, the mechanism of Hirano body formation is unknown.

Models to study Hirano bodies in living cells were developed by expressing altered forms of a 34 kDa *Dictyostelium* actin-binding protein in *Dictyostelium* and mammalian cell lines (Davis et al., 2008; Maselli et al., 2002, 2003) and mice (Furgerson et al., 2014; Ha et al., 2011b). These altered forms exhibit gain-of-function activated F-actin binding (Griffin et al., 2014; Maselli et al., 2002, 2003). Proteins present in Hirano bodies in postmortem tissues were also found in model Hirano bodies (Davis et al., 2008; Furgerson et al., 2012; Ha et al., 2011a; Maselli et al., 2002, 2003; Spears et al., 2014). These results prompted further investigation of some proteins found in brain specimens utilizing modern reagents in live cells expressing model Hirano bodies and have shed light on the possible physiological role(s) of Hirano bodies in neurodegenerative diseases. The presence of model Hirano bodies protected cells from death induced by AICD (intracellular domain of the amyloid precursor protein) (Furgerson et al., 2012; Ha et al., 2011a). The presence of model Hirano bodies and AICD and/or various forms of tau either protected cells from cell death or enhanced cell death depending whether the form of tau had a propensity to aggregate through enhanced phosphorylation (Spears et al., 2014). Since valuable information about the physiological role of Hirano bodies was obtained by re-examining proteins found to colocalize to Hirano bodies, we have developed a partial purification of model Hirano bodies and utilized mass spectrometry to elucidate the protein composition of model Hirano bodies in the model organism *Dictyostelium*. From the results of mass spectrometry, we focused on the role of proteins that regulate actin polymerization. The results support the hypothesis that *de novo* actin polymerization is required for model Hirano body formation.

## RESULTS

Model Hirano bodies in *Dictyostelium* are large (1-3 µm) F-actin-rich inclusions, readily detected by labeling with TRITC-labeled phalloidin. This property was utilized to follow their purification and enrichment by sedimentation following cell lysis and density gradient fractionation. Purification of model Hirano bodies to homogeneity was not possible due to the temporal instability of the model Hirano bodies after cell lysis. Observation of model Hirano bodies labeled with TRITC-phalloidin with time yielded a total time of approximately 1 h from cell lysis until the model Hirano bodies completely disassembled. Fractions from the Opti-prep gradient with model Hirano bodies also contained particles that were stained with DAPI, a general DNA fluorescent marker. Thus, it was expected to identify contaminant proteins/particles from mass spectrometry that localize to the nucleus and/or mitochondria that contain DNA.

Identification of approximately 135 proteins with two or more fragmented peptides was achieved; of these proteins, 37 had predicted sequences identifying them as ribosomal, 13 were proteasome components, 34 were mitochondrial proteins, 33 were proteins found in the cytoplasm and 18 were identified as linked to other pathways (Appendix Table A2). These proteins were a compilation of five runs. Approximately 270 proteins were identified with a single fragmented peptide. Several of these proteins (66) had been previously identified with two or more fragmented peptides. Several proteins comprise components of previously identified contaminants such as the mitochondria (28 proteins), ribosomes (34), endosomes (13), and proteasome (10). There were several putative and hypothetical proteins (27) and a variety of cytoplasmic proteins (52) identified. There were also 21 cytoskeletal proteins identified. Some of these proteins had been previously identified with multiple fragments, or in multiple mass spectrometry analysis, or that were subunits of proteins with multiple polypeptides such as myosin II. We investigated several of the cytoskeleton-associated proteins due to their role in actin polymerization (see below).

### Mitochondria do not colocalize with model Hirano bodies

To verify whether mitochondria are in model Hirano bodies or whether they were contaminants in the fraction containing them, we induced the expression of E60K-GFP (E60K-34 kDa protein fused to GFP, see Table S1) using the discoidin promoter for 24 h. The cells were stained with MitoTracker^®^ Red CMXRos (Invitrogen, Carlsbad, CA), a live cell dye, and fixed. The mitochondria did not colocalize with model Hirano bodies in fixed cells (Fig. S1). Thus, mitochondria and its associated proteins identified by mass spectrometry appear to be contaminants in the model Hirano body purification. All mitochondrial proteins were eliminated from the list of possible proteins in model Hirano bodies identified by mass spectrometry.

### The role of profilin I in model Hirano body formation

**Fig. 1.**
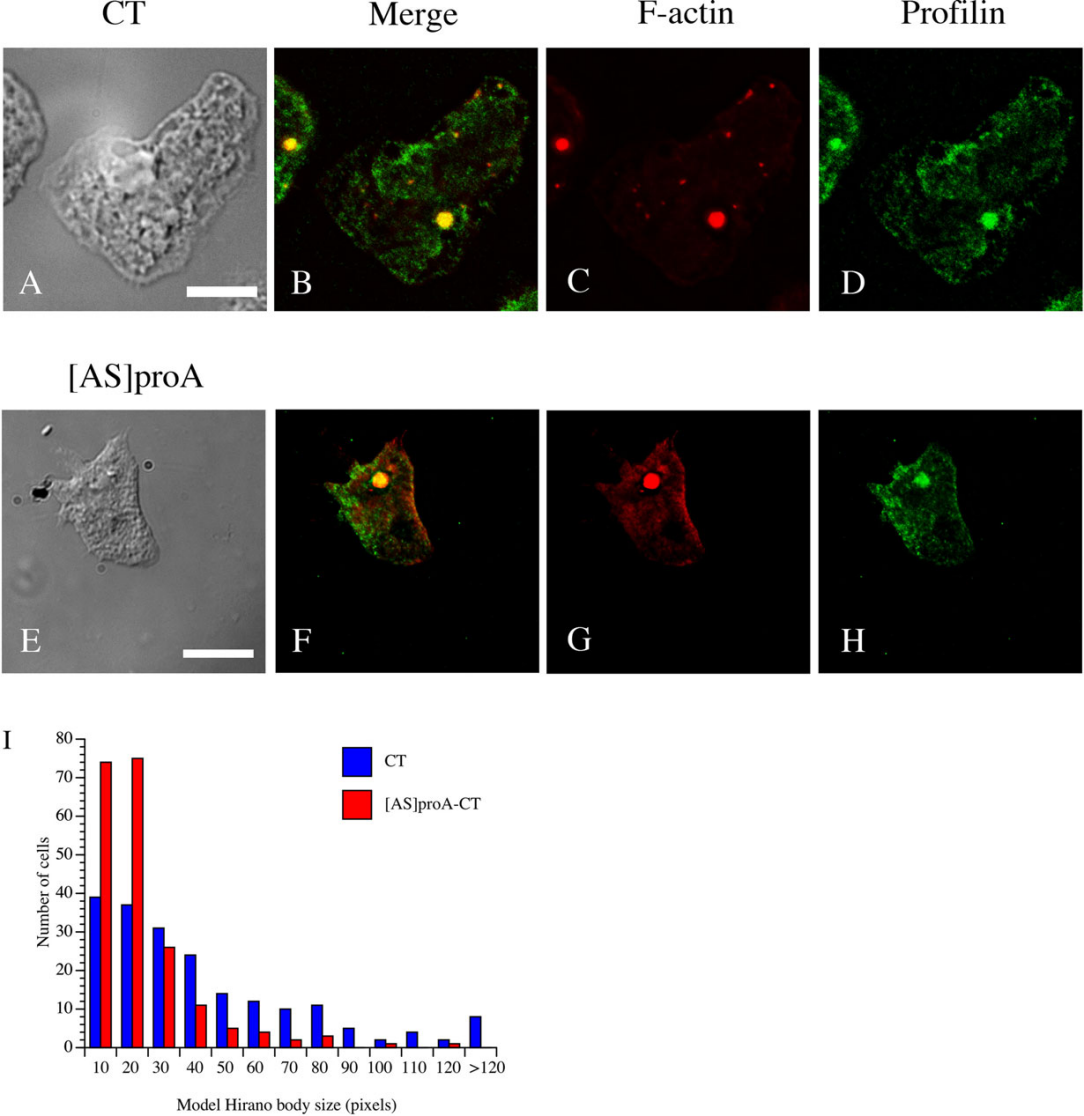
**Localization of Profilin I and F-actin in *Dictyostelium* cells expressing model Hirano bodies.** (A-H) Cells expressing CT or profilin knockdown strain [AS]proA transformed with CT were observed by DIC microscopy (A,E), and by fluorescence microscopy using either TRITC-phalloidin to stain F-actin (C,G) or antibody against profilin I (D) or B2C that recognizes CT (H), followed by FITC-conjugated secondary antibody. Profilin I is enriched in model Hirano bodies (B) and in cells with reduced levels of profilin (F). Scale bar=10 µm. (I) Profilin I affects the size of model Hirano bodies. The size of model Hirano bodies were measured in profilin knockdown strain [AS]proA-CT and CT cells in three independent experiments. A representative plot is shown. The size of model Hirano bodies significantly decreased when profilin I was knocked down (*P*<0.001 for all three experiments).

Using inducible promoters, it has been observed that model Hirano bodies begin as small actin foci that coalesce to form a large inclusion (Griffin et al., 2014; Reyes et al., 2009). It is not known whether the foci disassemble and reassemble to form the large inclusion or whether the foci coalesce into one inclusion. Upon examination of the mass spectrometry analysis, we observed that profilin and Arp2/3 were putatively present in model Hirano bodies. We further examined these proteins since they are important in the regulation of actin filament formation. We examined the colocalization of profilin I with model Hirano bodies in *Dictyostelium* expressing CT (c-terminal portion of 34 kDa protein, amino acids 129-295, see Table S1) by immunofluorescence. Profilin I was enriched and colocalized well with the model Hirano bodies (Fig. 1A-D). This result verified the presence of profilin I in model Hirano bodies. To determine if profilin I affects model Hirano body formation, profilin I knockdown strain ([AS]proA) (Haugwitz et al., 1994) was transformed with plasmid encoding CT under a constitutive promoter (Maselli et al., 2002). Transformed cells ([AS]proA-CT), [AS]proA and wild-type cells expressing CT were immunoblotted to confirm that expression of profilin I was reduced (data not shown). Model Hirano bodies still formed in transformed cells (Fig. 1E-H); however, compared with wild-type cells that expressed CT, model Hirano bodies that were formed when profilin I was knocked down were significantly smaller (I, *P*<0.001). This result suggests that profilin I promotes model Hirano body formation.

### Colocalization of the Arp2/3 complex with model Hirano bodies

**Fig. 2.**
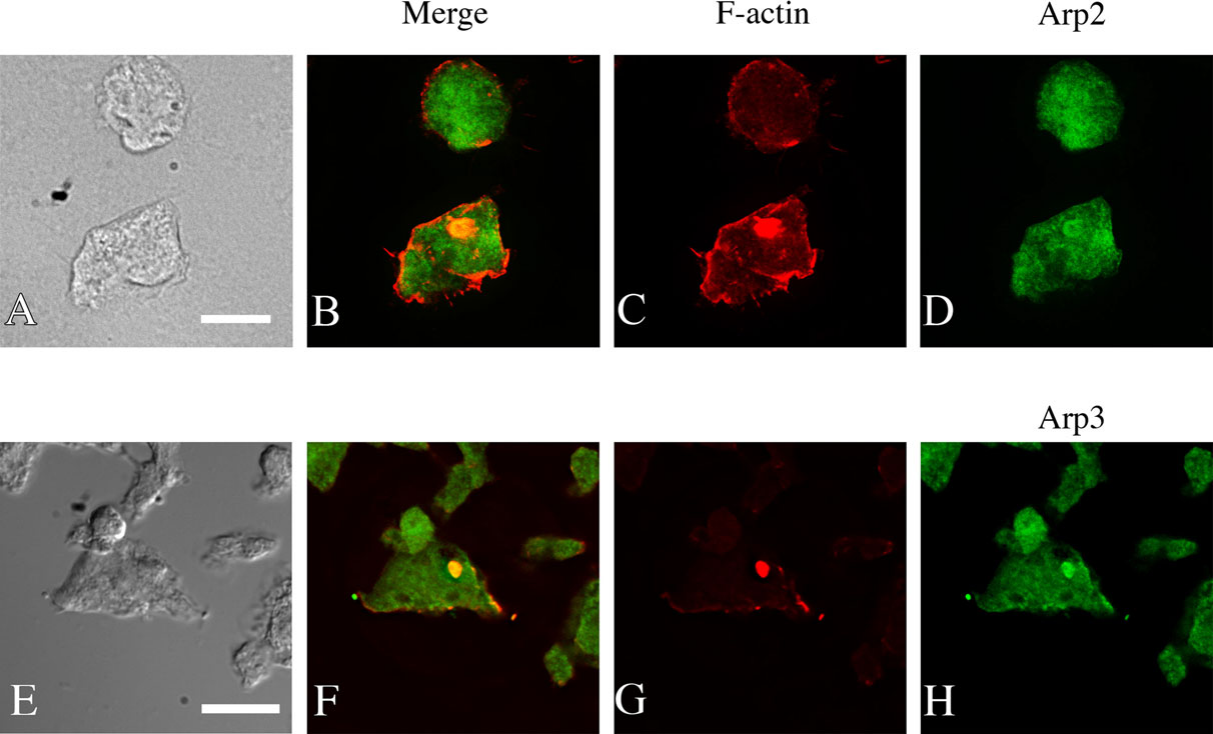
**Localization of Arp2 and Arp3 and F-actin in *Dictyostelium* cells expressing model Hirano bodies.** Cells expressing CT were transformed with plasmid to express GFP-Arp2 (A-D) or cells expressing GFP-Arp3 were transformed with CT (E-H). The cells were observed by DIC microscopy (A,E), and by fluorescence microscopy using either TRITC-phalloidin (C,G) to visualize F-actin, or GFP to visualize Arp2 and Arp3 (D,H). Arp2 and Arp3 are enriched in cortical actin-rich structures and also model Hirano bodies generated by expressing CT (B,F). Scale bar=10 µm.

The subunits of the Arp2/3 complex Arp3 and p21 (also named ARPC3) were identified by one peptide fragment each in the mass spectrometry analysis. Since Arp2/3 complex has an important regulatory role in actin polymerization, the presence of Arp2 and Arp3 in model Hirano bodies was tested to verify these results. *Dictyostelium* with CT constitutively expressed were transformed with plasmid encoding GFP-Arp2 (Insall et al., 2001) and stained with TRITC-labeled phalloidin. To also determine the localization of Arp3 compared to the model Hirano bodies, *Dictyostelium* constitutively expressing GFP-Arp3 (Insall et al., 2001) were transformed with plasmid encoding CT and stained with TRITC-labeled phalloidin. GFP-Arp2 was enriched in model Hirano bodies (Fig. 2A-D). GFP-Arp3 localized at similar regions of cells as GFP-Arp2, and was enriched in model Hirano bodies (Fig. 2E-H). These results support that the Arp2/3 complex colocalizes with model Hirano bodies, providing confirmation of the mass spectrometry data.

### Inhibition of the Arp2/3 complex activity inhibits model Hirano body formation

To determine the role of the Arp2/3 complex in model Hirano body formation, we utilized an Arp2/3 complex inhibitor, CK666, since the knockout of either Arp2 or Arp3 is lethal. It has been shown that CK666 specifically inhibits the actin nucleation activity of the Arp2/3 complex (Nolen et al., 2009). A mechanism was proposed that CK666 inhibits Arp2/3 complex activity by preventing a conformational reorganization crucial for activation (Hetrick et al., 2013); however it has never been used in *Dictyostelium*. In addition, the previous studies utilizing CK666 (Nolen et al., 2009; Yang et al., 2012) incubated it with the cells for approximately 30 min; however it takes at least 3 h to see the small F-actin foci forming, and more than 6 h to see a significant number of model Hirano bodies with larger sizes (Griffin et al., 2014; Reyes et al., 2009). Therefore, it was necessary to test whether CK666 inhibits the *Dictyostelium* Arp2/3 complex for a length of time comparable to model Hirano body formation before utilizing it to investigate the role of Arp2/3 complex in model Hirano body formation.

**Fig. 3.**
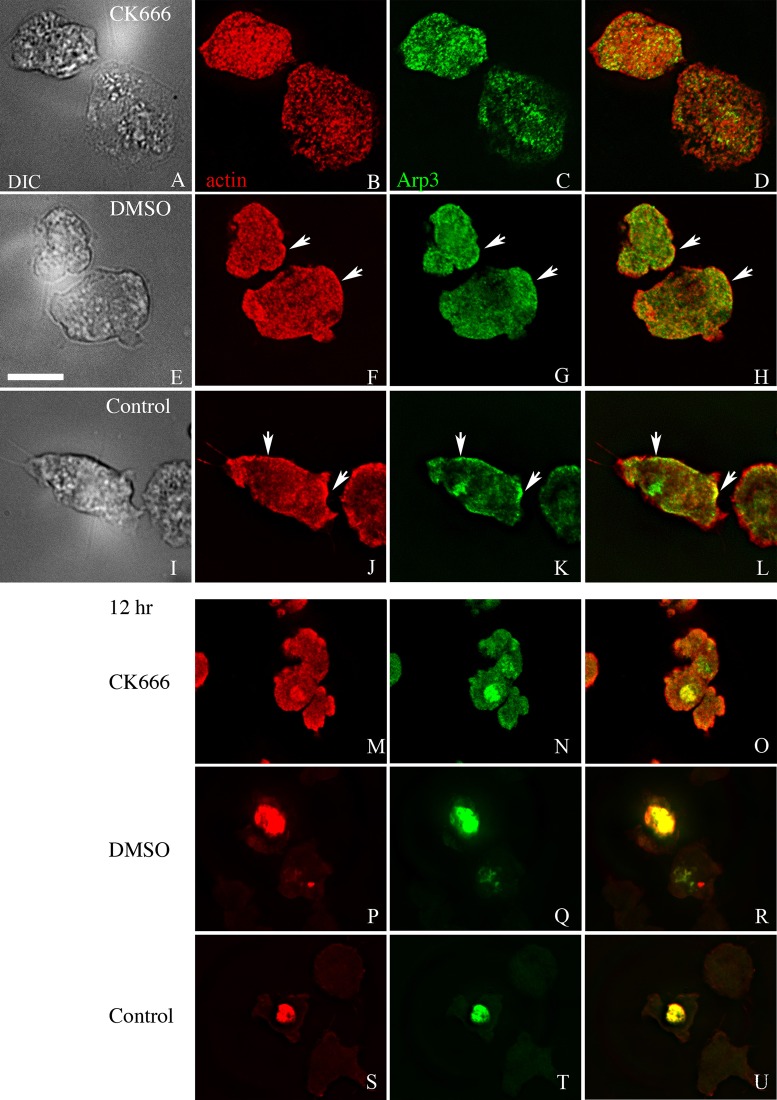
**CK666 disrupts the localization of Arp3 and F-actin.** GFP-Arp3-expressing cells were incubated in the presence of either CK666 in media (A-D), or DMSO in media (E-H), or media only (I-L) for 18 h and examined by DIC microscopy, and by fluorescence microscopy using TRITC-phalloidin (second column) or GFP (third column). GFP-Arp3 is enriched at the cortex and lamellipodia, colocalizing with F-actin, in the cells with DMSO in media or media only. In contrast, this colocalization is disrupted in cells with media plus CK666. Arrows indicate the enrichment of GFP-Arp3 and F-actin. (M-U) Model Hirano body formation is inhibited by CK666. Cells were induced to express E60K-GFP, and were immediately incubated for 12 h with CK666 in media (M-O), DMSO in media (P-R), or media only (S-U) and examined by fluorescence microscopy using TRITC-phalloidin (left column) or GFP (middle column). Compared with the controls, E60K-GFP (N) and F-actin (M) appear less focused when incubated in media plus CK666. Scale bar=10 µm.

To determine if CK666 inhibits *Dictyostelium* Arp2/3 complex activity and the time span of efficacy, *Dictyostelium* cells constitutively expressing GFP-Arp3 were incubated with 100 µM CK666, with 0.2% DMSO/media as the solvent control, or media only, for different lengths of time, followed by fixation and staining with TRITC-labeled phalloidin to localize F-actin. In agreement with published data, GFP-Arp3 colocalized with F-actin at the cortex and the lamellipodia in the controls (Fig. 3G,K) (Insall et al., 2001). In contrast, in the cells incubated in the presence of CK666, the colocalization of F-actin and GFP-Arp3 was reduced and GFP-Arp3 was not localized in the cortex or lamellipodia (Fig. 3C). This shows that CK666 inhibited the activity of the Arp2/3 complex in *Dictyostelium*, an effect lasting for at least 18 h. Therefore, CK666 could be used as a tool to examine the role of the Arp2/3 complex in model Hirano body formation.

**Fig. 4.**
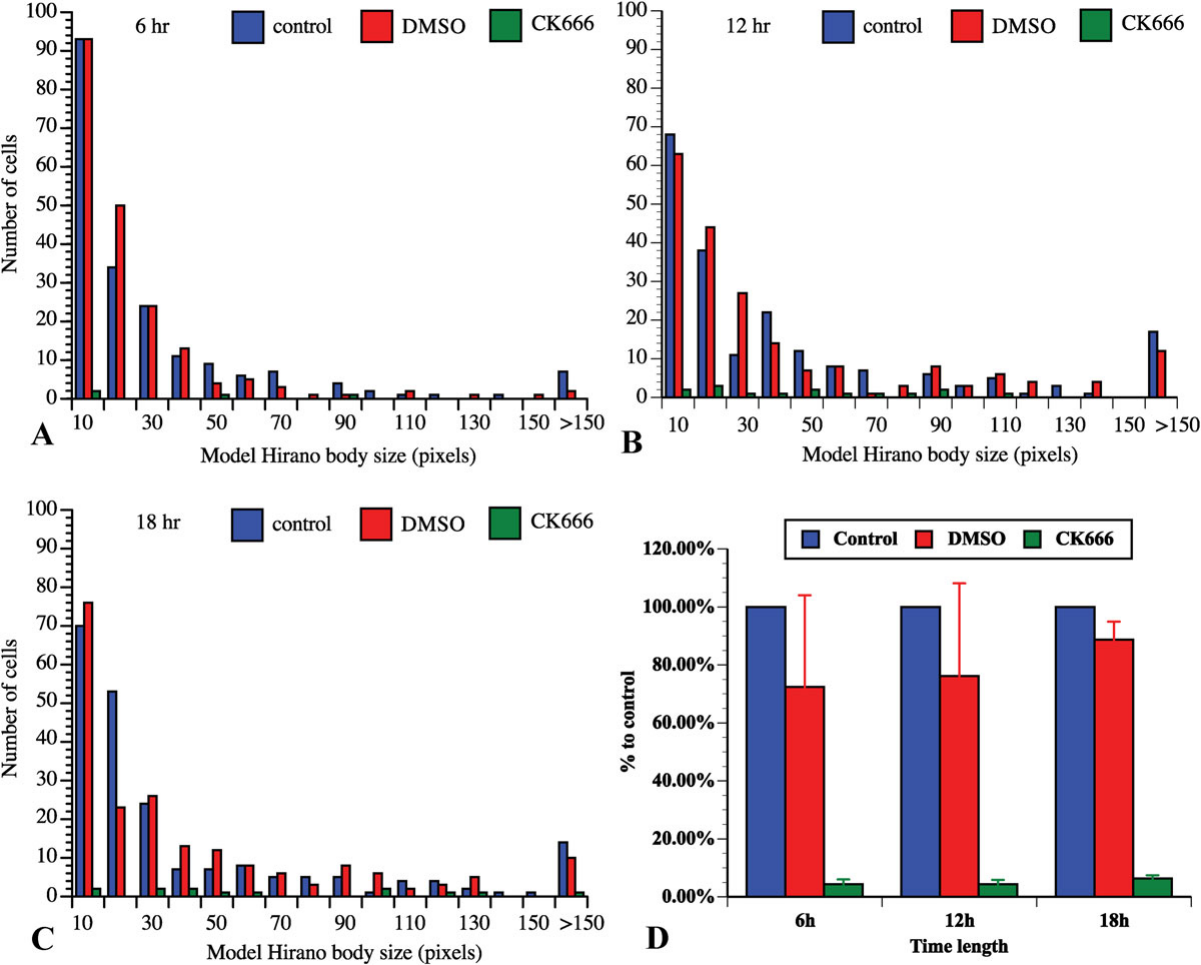
**CK666 affects the distribution of model Hirano body size and number of cells that generate model Hirano bodies.** The size distribution of model Hirano bodies was measured after 6 h (A), 12 h (B) or 18 h (C) of incubation with CK666 in media (green) after removal of folic acid to induce expression of E60K-GFP, compared with cells incubated with DMSO plus media (red) or media only (blue) under the same condition to induce model Hirano bodies. The distributions of the model Hirano body size were significantly different (*P*<0.01 at 6 h, *P*<0.001 at 12 h and at 18 h). The proportions of cells that generated model Hirano bodies with or without CK666 were also counted, and compared with the control (D). The number of cells that produced model Hirano bodies were significantly reduced when incubated in the presence CK666 (*P*<0.001 for all three time points, data represented as means±S.D.).

Subsequently, E60K-GFP cells were induced in the presence or absence of 100 µM CK666. Cells were fixed and stained with TRITC-phalloidin to visualize the F-actin at 6 h, 12 h and 18 h after induction of model Hirano bodies (12 h shown in Fig. 3M-U). The cells in the presence of CK666 formed fewer model Hirano bodies than control cells, consistent with the data that Arp2/3 colocalized with model Hirano bodies. Some of the model Hirano bodies that formed in the presence of CK666 showed abnormal morphology compared to the controls. To quantify these observations, the proportion of cells with model Hirano bodies, and the size of model Hirano bodies was counted in each of the samples. At each of the time points, compared with the controls, the proportion of cells that formed model Hirano bodies was dramatically lowered by 90% (Fig. 4, *P*<0.001 for all three time points). Moreover, the distribution of area of model Hirano bodies was significantly different compared to solvent control, (*P*<0.01 at 6 h, *P*<0.001 at 12 h and at 18 h; Fig. 4). These findings were repeated in three independent experiments.

**Fig. 5.**
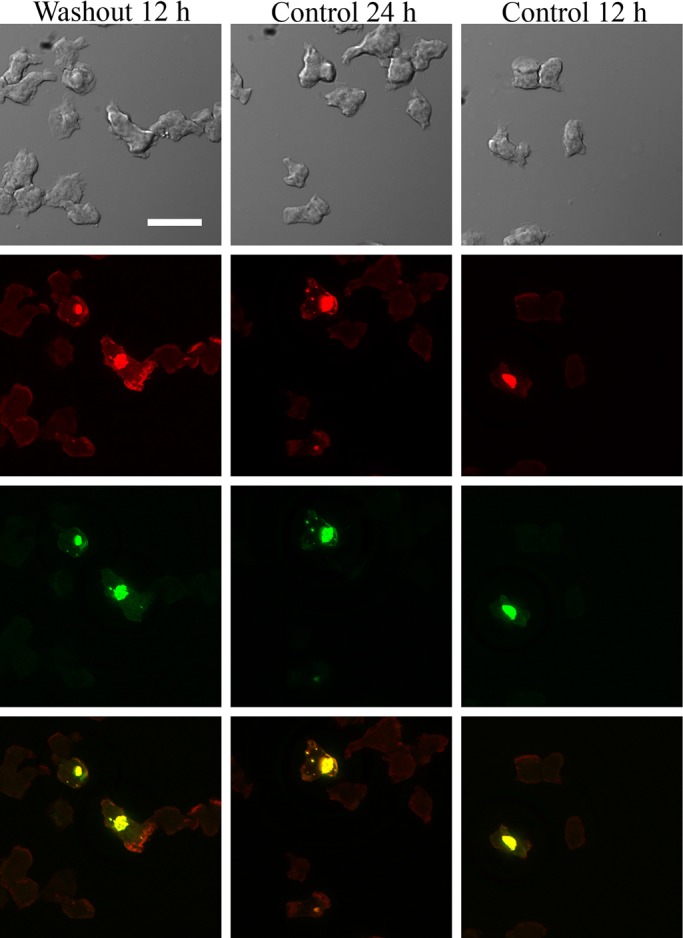
**Model Hirano bodies form after removal of CK666.** Cells were induced to express E60K-GFP and immediately incubated with CK666 for 12 h. They recovered in media after CK666 was removed for another 12 h (left column). Cells that were induced to express E60K-GFP at the same time (middle column) and at the time of CK666 removal (right column) were also observed as controls. All cells were examined by DIC (first row), and by fluorescence microscopy using TRITC-phalloidin (second row) or GFP (third row). Model Hirano bodies formed in a substantial amount of cells in all three samples. Scale bar=15 µm.

To rule out the possibility that the inhibition of model Hirano body formation by CK666 was due to a non-specific inhibition, we removed CK666 after 12 h of incubation and allowed the cells to recover for another 12 h with media under conditions for expression of E60K-GFP. Following removal of CK666, model Hirano bodies formed in a significant proportion of cells (Fig. 5). Thus, the effect of CK666 is reversible, and the process of model Hirano body formation can still be triggered. Therefore CK666, which inhibits the Arp2/3 complex activity, also inhibits model Hirano body formation and confirms that Arp2/3 is required for model Hirano body formation in *Dictyostelium*.

### Colocalization of the activators of the Arp2/3 complex, HSPC300 and WASH, with model Hirano bodies

**Fig. 6.**
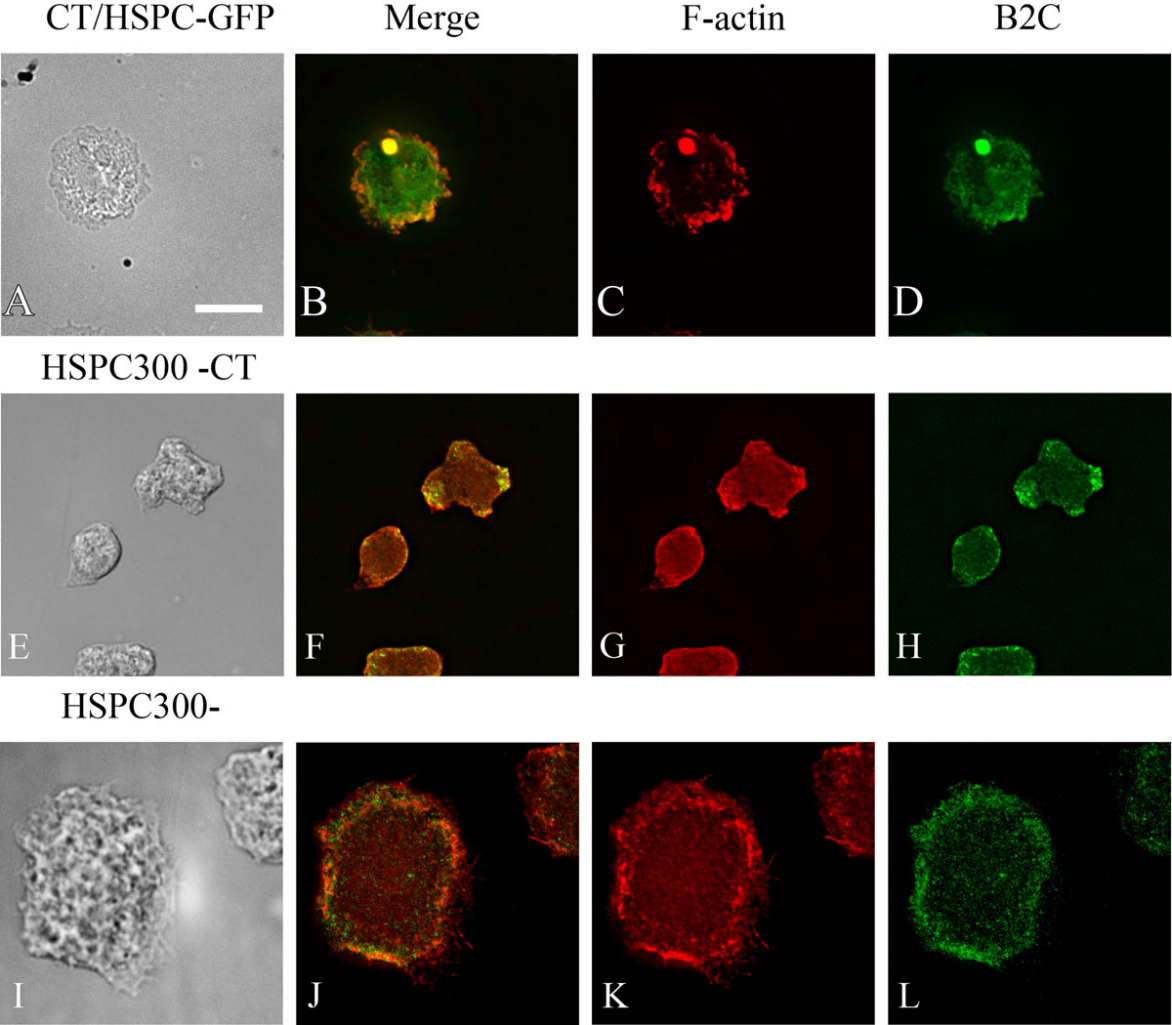
**Localization of HSPC300 and F-actin in *Dictyostelium* cells expressing model Hirano bodies.** (A-D) Cells expressing CT were transformed with plasmid to express HSPC300-GFP, and were observed by DIC microscopy (A), and by fluorescence microscopy using either TRITC-phalloidin (C) or GFP (D). HSPC300 is enriched in model Hirano bodies (B,D). (E-L) HSPC300 is required for model Hirano body formation. HSPC300^−^ transformed with CT (E-H), and control HSPC300^−^ (I-L), were examined by DIC microscopy (E,I), and by fluorescence microscopy using TRITC-phalloidin (G,K) or B2C that recognizes CT (H,L). No model Hirano bodies were observed. Scale bar=10 µm.

**Fig. 7.**
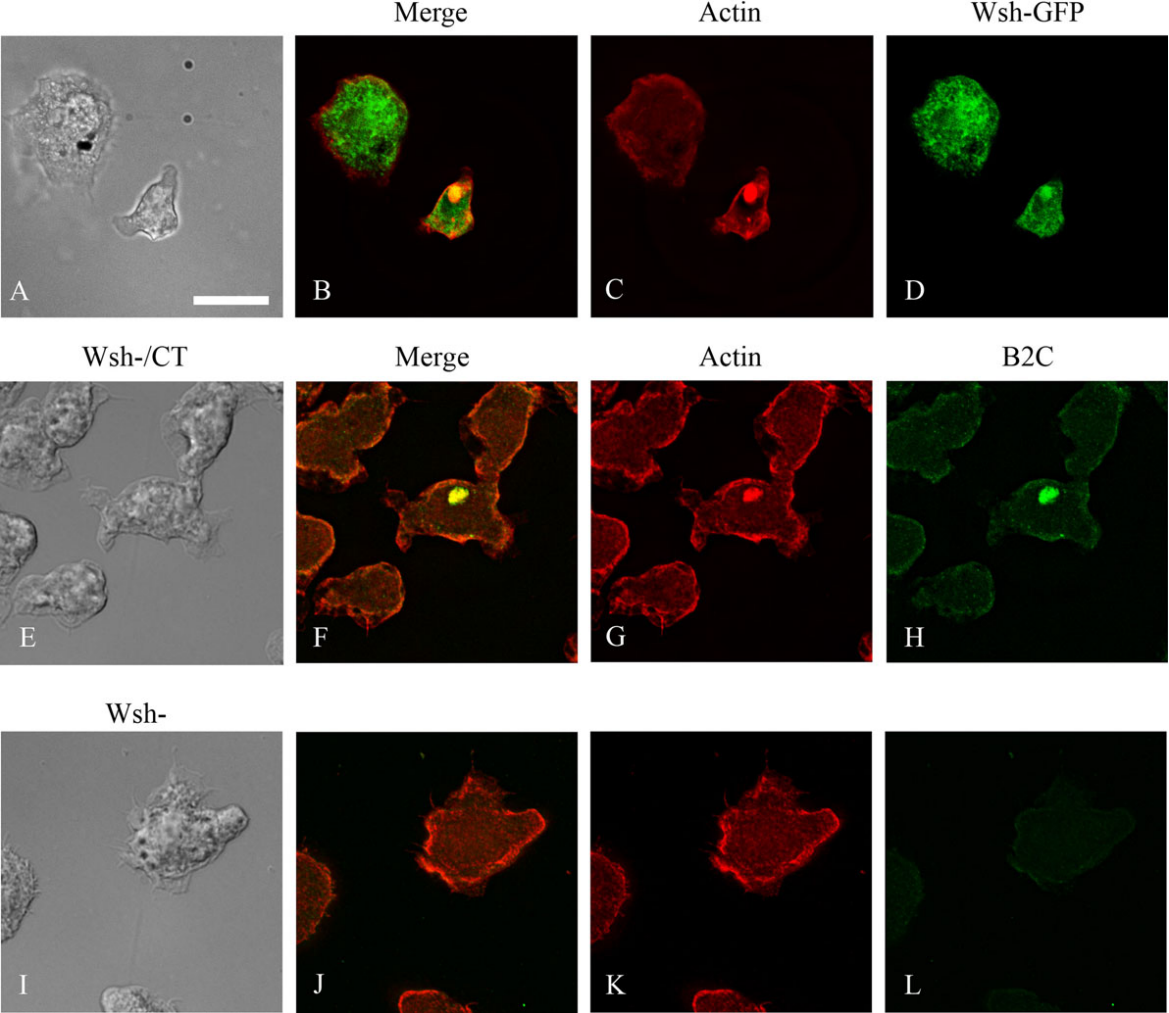
**Localization of WASH and F-actin in *Dictyostelium* cells expressing model Hirano bodies.** (A-D) Cells expressing CT were transformed with plasmid to express WshA-GFP, and were observed by DIC microscopy (A), and by fluorescence microscopy using either TRITC-phalloidin (C) or GFP (D). WASH is enriched in model Hirano bodies (B,D). (E-L) WshA is not required for model Hirano body formation. WshA^−^ transformed with CT (E-H), and control WshA^−^ (I-L), were examined by DIC microscopy (E,I), and by fluorescence microscopy using TRITC-phalloidin to stain F-actin (G,K) or B2C that recognizes CT (H,L). Model Hirano bodies enriched with CT were found in transformed cells. Scale bar=10 µm.

The Arp2/3 complex promotes nucleation of actin monomers to polymerize into filamentous actin. The Arp2/3 complex by itself has weak activity and requires activation for maximal activity. One of the contributors for activation is interaction with nucleation-promoting factors (NPFs) (Campellone and Welch, 2010). NPFs stimulate Arp2/3 nucleation activity through their carboxy-terminal WCA domains but have diverse amino-terminal domains, allowing for differential cellular function and regulation. We examined the localization of two of the activators of the Arp2/3 complex, HSPC300, part of the WAVE complex, crucial to plasma membrane protrusion and cell motility; and WASH, which is involved in endosome trafficking to ascertain whether they also colocalized with model Hirano bodies. Cells constitutively expressing CT were transformed separately with either HSPC300-GFP (Pollitt and Insall, 2009) or WASH-GFP (Carnell et al., 2011) and were subsequently stained with TRITC-labeled phalloidin to visualize F-actin. Cells transformed with either HSPC300-GFP or WASH-GFP showed localization analogous to Arp2 or Arp3 (Fig. 6A-D, Fig. 7A-D) and were also enriched in model Hirano bodies. Together, these results suggest that model Hirano body formation involves the activity of the Arp2/3 complex, and its activation by the nucleation polymerization factors including WASH and wave regulatory complex (WRC), with HSPC300 as a subunit. The roles of these proteins in model Hirano body formation were further investigated.

### The role of HSPC300 and WASH in model Hirano body formation

Since HSPC300 and WASH colocalized with model Hirano bodies, knockout strains of HSPC300 and WASH were used to further test the result that the Arp2/3 complex plays a part in model Hirano body formation, and indicate a possible pathway how the Arp2/3 complex is activated in model Hirano body formation. To determine if model Hirano bodies form in HSPC300^−^, HSPC300^−^ (Pollitt and Insall, 2009) were transiently transformed with plasmid encoding CT under a constitutive actin promoter. Cells were fixed at different times after transformation with the result that no model Hirano bodies were observed (Fig. 6E-H). This indicates that HSPC300, and the WRC, is critical for model Hirano body formation through the regulation of the Arp2/3 complex. In contrast, when WASH^−^ (Carnell et al., 2011) was transiently transformed with plasmid encoding CT, actin inclusions formed in a substantial proportion of cells (Fig. 7E-H). These inclusions also colocalized with CT, indicating that they are model Hirano bodies and that WASH is not required for formation of model Hirano bodies.

### The role of VASP in model Hirano body formation

**Fig. 8.**
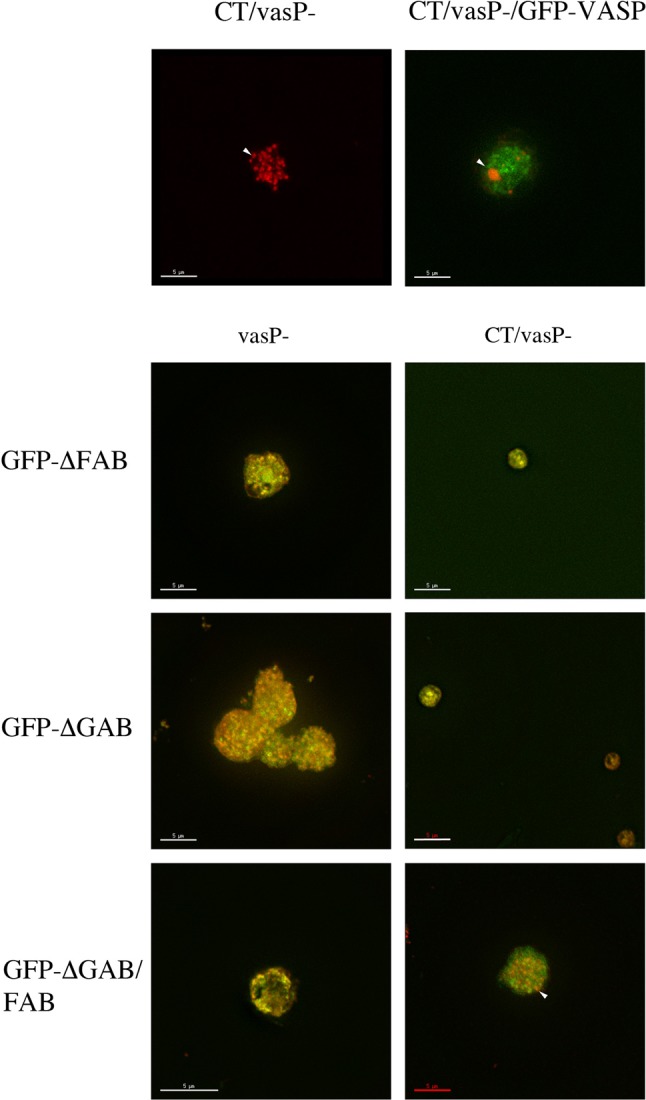
**Localization of VASP and mutant forms of VASP and F-actin in *Dictyostelium* cells transformed with CT to induce model Hirano bodies.** vasP^−^ were transformed with plasmid to express GFP-VASP, GFP-ΔGAB, GFP-ΔFAB, GFP-ΔGAB/FAB and in the presence or absence of CT to induce model Hirano bodies. The cells were observed by fluorescence microscopy using either TRITC-phalloidin or GFP. Except for CT/vasP^–^, the merged TRITC-phalloidin and GFP images are shown. When CT is expressed in vasP^−^, punctate small actin foci were formed. Model Hirano bodies were formed when CT/vasP^−^ was rescued with full length GFP-VASP. Small foci containing either actin, VASP, or both were observed when either GFP-ΔGAB or GFP-ΔFAB VASP was expressed in vasP^−^. In the presence of CT and either GFP-ΔGAB or GFP-ΔFAB VASP, no large model Hirano bodies were formed, only small foci. When CT is expressed in GFPΔGAB/FAB vasP^−^, punctate actin foci that resembled those formed in vasP^–^ cells were observed. VASP and its ability to bind actin is required either for F-actin foci to coalesce or elongate from F-actin foci to form model Hirano bodies. Arrowheads indicate either actin foci or model Hirano bodies. Scale bar=5 µm.

Since profilin and the actin nucleation factor Arp2/3 have been shown to be important for model Hirano body formation, we also studied the effect of VASP, an actin elongation factor. To determine whether model Hirano bodies require VASP to form, *Dictyostelium* lacking VASP (vasP^−^) (Han et al., 2002) were transformed with CT to induce model Hirano bodies in the presence or absence of full length VASP, VASP lacking either the G- (ΔGAB) or F-actin binding domains (ΔFAB), or both the G- and F-actin binding domains (ΔGAB/FAB) (Breitsprecher et al., 2008; Schirenbeck et al., 2006). Transient expression in cells was determined with TRITC-labeled phalloidin to localize actin and GFP fluorescence was used to localize VASP and its mutants as shown in Fig. 8. No model Hirano bodies formed in CT/vasP^−^ cells, only numerous punctate F-actin foci were observed. In CT/VASP/vasP^−^ cells, model Hirano bodies formed and VASP did not colocalize to the model Hirano body. VasP^−^ cells transformed with VASP lacking either the ΔGAB or ΔFAB formed small punctuate foci that were enriched in either actin, VASP, or both (Fig. 8). When CT is expressed in these cells, no model Hirano bodies form, only small foci formed as seen in the control cells. The phenotype of numerous, small punctate actin foci in the CT/vasP^−^ cells was recovered when ΔGAB/FAB VASP was expressed. While VASP does not colocalize to model Hirano bodies, it is required for model Hirano body formation.

## DISCUSSION

Model Hirano bodies have been induced in *Dictyostelium*, mammalian cultured cells, and a range of complex physiological effects have been reported (Davis et al., 2008; Furgerson et al., 2012; Ha et al., 2011a; Maselli et al., 2002, 2003; Spears et al., 2014). These results led to the effort to identify the proteins in these inclusions. The model organism *Dictyostelium* was chosen for this study since it is facile and cost effective to grow large quantities of cells for biochemical purification, has a curated genome, many reagents in the form of plasmids and mutant strains available, and facile genetics to create new strains. Model Hirano bodies were partially purified from *Dictyostelium* cells using Opti-prep density gradients and sedimentation. A variety of dense particles including mitochondria, ribosomes, and the vault particle were also recovered at the same density as model Hirano bodies. The samples were not further purified due to the instability of model Hirano bodies. The components of model Hirano bodies were identified by mass spectrometry and included cytoskeletal, ribosomal, and mitochondrial proteins (Appendix Table A2). Since model Hirano bodies were not purified to homogeneity, immunofluorescence was utilized to verify the presence of proteins/organelles identified by mass spectroscopy. We verified that mitochondria do not colocalize with model Hirano bodies (Fig. S1). Ribosomes are probably contaminants as well but this is difficult to confirm due to lack of suitable reagents. Components of the proteasome were also identified by mass spectrometry. The presence of proteasomes could be contaminants or could have been associated with model Hirano bodies undergoing degradation (Kim et al., 2009).

Since the major protein component of Hirano bodies is filamentous actin (Goldman, 1983) and actin binding proteins have been previously localized to Hirano bodies (Davis et al., 2008; Maciver and Harrington, 1995; Maselli et al., 2002), we focused on the actin regulatory proteins identified by mass spectrometry. Actin binding proteins, myosin II heavy and light chains, α-actinin, Arp2/3 complex, profilin I and II, hsp70, fimbrin, coactosin, EF1 alpha, vinculin-related protein, histophilin II, cortexillin I, ABP1 and filamin were identified. The presence of α-actinin, myosin II (Maselli et al., 2002), Arp2/3 complex and profilin I in model Hirano bodies were confirmed; however, filamin and EF1 alpha were previously found not to colocalize to model Hirano bodies (Maselli et al., 2002). Thus, it is important to confirm results obtained by mass spectrometry analysis by immunofluorescence.

The role of profilin I and Arp2/3 complex were chosen for further investigation due to their role in the regulation of F-actin polymerization. These proteins were localized to model Hirano bodies (Figs 1, 2). Verification of the role of profilin I in the formation of model Hirano bodies was achieved utilizing a profilin knockdown strain. Our results show that model Hirano bodies are significantly smaller when profilin I is knocked down in *Dictyostelium* (Fig. 1I). The actin monomer binding protein profilin has been shown to increase the assembly rate of actin filaments by facilitating the exchange of ATP for ADP on G-actin, maintaining the ATP-G-actin pool that polymerizes more readily than ADP-G-actin (Perelroizen et al., 1996). Together, this suggests that profilin-enhanced assembly of F-actin is critical to the size of the model Hirano bodies, highlighting the role of *de novo* actin polymerization in model Hirano body formation.

Verification of the role of the Arp2/3 complex in the formation of model Hirano bodies was achieved utilizing the small molecule inhibitor CK666 (Fig. 3), again suggesting that *de novo* actin polymerization is required. Hirano bodies exhibit a hallmark paracrystalline order observed by electron microscopy. The appearance of the Hirano body depends on the plane of section and can vary from a lattice to a herringbone pattern (Schochet and McCormick, 1972; Tomonaga, 1974). Both Hirano bodies from mammalian sources (Peterson et al., 1986) and *Dictyostelium* (Maselli et al., 2002) were frequently observed to have a juxtaposition of ordered and disordered regions. The Arp2/3 complex nucleates F-actin by binding at the sides of preexisting actin filaments, initiating actin filament branches at an angle of approximately 70° from the mother filament with Arp2 and Arp3 as the first two subunits of the daughter filament (Mullins et al., 1998; Rouiller et al., 2008). The resultant F-actin branched structure could be a contributing factor to the changing appearance of the Hirano body with the plane of section.

Identification of the pathway(s) of Arp2/3 activation was investigated through knockout strains of HSPC300 (Caracino et al., 2007), a component of the WAVE complex which activates Arp2/3 in actin polymerization responsible for membrane protrusion and cell motility, and WASH which activates Arp2/3 in the endocytic pathway (Carnell et al., 2011). No model Hirano bodies were found in transformed HSPC300^−^ cells (Fig. 6), but were found in transformed WASH^−^ cells (Fig. 7). This suggests that the WAVE regulatory complex (WRC), of which HSPC300 is a subunit, contributes to model Hirano formation through the activation of the Arp2/3 complex. WASH associates with model Hirano bodies and was shown to play an important role in exocytosis and vesicle trafficking. Model Hirano bodies are cleared from cells by autophagy (Kim et al., 2009) and WASH may play a role in the degradation pathway of Hirano bodies. Together with the results obtained from the profilin knockdown cells, the data suggests that *de novo* actin polymerization is required for model Hirano body formation.

The WRC also needs to be activated to function. Its main activator and recruiter Rac1 (Chen et al., 2014; Eden et al., 2002) could be potentially involved in the pathway to form model Hirano bodies. It was previously shown that increased Rac1 activity is associated with the enhancement of actin polymerization induced by fibrillar amyloid beta peptide (Mendoza-Naranjo et al., 2007). Moreover, WAVE was shown surrounding senile plaque in postmortem tissue, upregulated in tissues from AD patients, and colocalized with tau in AD mouse models (Kitamura et al., 2003; Takata et al., 2009). Tau is also colocalized to Hirano bodies (Galloway et al., 1987b; Peterson et al., 1988; Spears et al., 2014). Taken together, this suggests that the formation of Hirano bodies could be promoted by the pathology of neurodegeneration and that the pathway involves WRC and the Arp 2/3 complex, and possibly Rac1.

VASP was found to be required for model Hirano body formation although it was not colocalized to them. The small actin-rich structures observed in CT/vasP^–^ and CT/vasP^–^/GFP-VASPΔGAB/FAB indicate that both the bundling and actin elongation activities are required for model Hirano body formation. CT/vasP^–^/GFP-VASPΔFAB cells failed to form model Hirano bodies. Previous investigation of *Dictyostelium* VASP revealed that DdVASPΔFAB could not rescue filopodia formation in vasP^−^ cells (Schirenbeck et al., 2006) nor bundle F-actin (Schirenbeck et al., 2006) but mediated accelerated filament elongation (Breitsprecher et al., 2008). These results are consistent with the observation that model Hirano bodies require both the elongation and bundling activities of VASP; however, DdVASPΔGAB did rescue filopodia formation and accelerate filament elongation (Breitsprecher et al., 2008). Curiously, model Hirano bodies failed to form in the presence of VASPΔGAB. Mutation in GAB site was reported to produce instability of filopodia tips (Applewhite et al., 2007). This stabilizing activity could be required *in vivo* for model Hirano body formation in addition to the bundling and filament elongation activities. The mutant DdVASPΔGAB/FAB did not bundle actin nor accelerate filament elongation (Breitsprecher et al., 2008) so it was not surprising that model Hirano bodies were not formed.

It has been reported that the crosslinking of filamentous actin (Griffin et al., 2014; Maselli et al., 2002, 2003), the transportation and clustering of small actin aggregations (Reyes et al., 2009), and the stabilization of filamentous actin (Kim et al., 2009; Lázaro-Diéguez et al., 2008) are critical for model Hirano body formation. The current results provide evidence that *de novo* actin polymerization regulated by the Arp 2/3 complex and profilin, is required for the formation of model Hirano bodies. VASP is necessary for enhanced filament elongation for large model Hirano bodies to form. Model Hirano bodies are stable, cytoplasmic inclusions comprised of filamentous actin and appear to be formed by the same proteins required for dynamic rearrangement of the actin–rich filopodia. It is interesting to note that the parental protein (ABP34) of the mutant forms that induced model Hirano body formation is highly enriched in the filopodia of *Dictyostelium*. It was postulated previously (Svitkina et al., 2003) that filopodia are formed from a network of filaments whose growth is mediated by Arp2/3. The barbed ends of some filaments are protected by a complex that includes VASP which provides the ability for further elongation and stabilization by the addition of crosslinking proteins such as fascin. This model is controversial. Nonetheless, the proteins that enable filopodia formation were those found to be essential for model Hirano body formation and suggests that some shared mechanism(s) contributes to model Hirano body formation. The stability of model Hirano bodies could be due to the activated actin-binding properties of the mutant proteins, and the high affinity and stoichiometry of binding to F-actin (Griffin et al., 2014; Lim et al., 1999b; Maselli et al., 2003). Furthermore, ABP34 has been shown to slow the rate of depolymerization of F-actin (Zigmond et al., 1992). Future investigation is needed regarding how and what the signal(s) is to initiate the polymerization and crosslinking of actin filaments into model Hirano bodies.

## MATERIALS AND METHODS

### *Dictyostelium* culture

All *Dictyostelium* strains except cells lacking VASP (vasP^−^) were grown and maintained as axenic cultures in liquid media supplemented with appropriate antibiotics (see Table S1) at 20°C and shaking at 150 rpm. *Dictyostelium* cells were grown to a maximum density of 1-2×10^6^ cells/ml. vasP^−^ were grown on tissue culture plates. For cells with the pVEII vectors (Blusch et al., 1992), the media was supplemented with 1 mM folate and grown to a maximum density of 1×10^6^ cells/ml, in order to repress the expression of E60K-GFP driven by the discoidin promoter. No cells were maintained for more than 4 weeks.

To induce E60K-GFP expression by the discoidin promoter in pVEII, 5×10^6^ cells were plated in a 100 mm petri dish, washed twice with 17 mM phosphate buffer, and grown with 10 ml HL-5 media supplemented with appropriate antibiotics at 20°C.

*Dictyostelium* cells were transformed with corresponding expression vectors (see Table S1) as previously described (Rivero et al., 1996). The plasmids GFP-Arp2 (Insall et al., 2001), HSPC300-GFP, and WASH-GFP (Park et al., 2013) were generous gifts from R. H. Insall (CRUK Beaton Institute, UK). The plasmids GFP-VASP, GFP-ΔGAB VASP, GFP-ΔFAB, GFP-ΔGAB/FAB VASP (Breitsprecher et al., 2008; Schirenbeck et al., 2006) were generous gifts from J. Faix (Hannover Medical School, Germany). The strains [AS]proA (Haugwitz et al., 1994), HSPC300^−^ (Pollitt and Insall, 2009), WASH^−^ (Carnell et al., 2011), and vasP^−^ were procured from the Dicty Stock Center (Northwestern University, IL) (Basu et al., 2013; Fey et al., 2013). G418 and blasticidin S were purchased from Life Technologies (Carlsbad, CA), and hygromycin B from EMD Millipore (Billerica, MA).

### Purification of Hirano bodies

The cells expressing CT and containing model Hirano bodies were harvested at 22°C at 1000 ***g*** for 5 min, resuspended, and washed twice in 17 mM phosphate buffer. The cells were resuspended to a concentration of 2×10^6^ cells/ml in a lysis buffer containing 5 mM Pipes, 5 mM EGTA, 1 mM MgCl_2_, 1 mM ATP, at pH 6.5 on ice. The cells were lysed by the addition of Brij detergent (Sigma-Aldrich, St. Louis, MO) to a final concentration of 0.5% in a Dounce homogenizer (Wheaton) and sheared with 10 strokes every 2 min for 16-18 min to guarantee total lysis of the cells. The cells were checked for lysis using light microscopy and a sample was taken for fluorescence microscopy. The lysed samples were sedimented at 1000 ***g*** for 5 min at 4°C. The pellet was resuspended to 150 µl with lysis buffer and samples were removed for fluorescence microscopy. The remaining sample was placed on an Opti-prep (Sigma-Aldrich, St. Louis, MO) step gradient. The Opti-Prep density gradient medium is a 60% solution of iodixanol in water, density=1.32 g/ml. Opti-prep step gradient was made using lysis buffer as a diluent as follows: 25%, 30%, and 50% Opti-prep. Samples were sedimented at 15,000 ***g*** for 20 min at 22°C in a swinging bucket rotor. The fractions from the density gradient were collected and stained using TRITC-labeled phalloidin and DAPI to assess the presence of Hirano bodies and nuclei, respectively. Fractions containing Hirano bodies were collected for mass spectrometry and frozen at −80°C.

### Mass spectrometry

For LC-MS/MS analysis, Hirano body preparations were denatured in urea, reduced, alkylated, and digested with sequencing grade trypsin (Promega, Madison, WI) following standard procedures (Wells et al., 2002). The resulting peptides were desalted using reverse-phase C18 spin columns (The Nest Group, Inc, Southborough, MA) and nitrogen bomb-loaded onto 0.075 mm×10 cm in-house packed C18 column/emitters (New Objective, Woburn, MA). Peptides were eluted for 2 h with a linear gradient of acetonitrile in 0.1% formic acid at a flow rate of approximately 250 nl/min into a linear ion trap (LTQ, ThermoElectron, Waltham, MA). One full ms scan followed by eight CID MS/MS scans of the most abundant ions were collected. Dynamic exclusion was set at 2. All data analysis was performed using TurboSequest (ThermoElectron) against the non-redundant Dictyostelium database (Eichinger et al., 2005; dictybase.org) as well as an inverted database to calculate false discovery rate (less than 1% in each run), and using stringent filtering of data. Fragments belonging to actin were excluded due to the abundance of actin in model Hirano bodies. Approximately 135 proteins were identified with two or more fragmented peptides; of these proteins, 37 had predicted sequences identifying them as ribosomal, 13 were proteasome components, 34 were mitochondrial proteins, 33 were proteins found in the cytoplasm and 18 were identified as linked to other pathways (Table S2). These assignments were based on a compilation of five independent runs.

### Data analysis

For protein identification, MS^n^ data were searched against translated genomic database (Eichinger et al., 2005; curated at dictybase.org) using SEQUEST (Bioworks 3.3, Thermo Fisher Scientific) with the following settings: 1000 ppm (10 ppm for data acquired using LTQ Orbitrap XL™) tolerance was set for precursor masses and 0.5 Da for fragment masses; trypsin was specified as the enzyme and only fully tryptic peptide identifications were retained; a maximum of three missed cleavage sites, three differential amino acids per modification, and three differential modifications per peptide were allowed; oxidization of methionine (+15.99 Da), carboxyamidomethylation of cysteine (+57.02 Da), phosphorylation of serine/threonine/tyrosine (+79.97 Da), and O-GlcNAc modification of serine/threonine (+203.08 Da) were set as differential modifications. All the raw spectra were searched against both normal (forward) and reverse databases under the same parameters. Peptides were filtered to 1% false-discovery rate and protein assignments were required to have a minimum of two independent peptides.

### Fluorescence microscopy

*Dictyostelium* cells were prepared for immunofluorescence to visualize specific proteins and/or structures as previously described (Fechheimer, 1987). F-actin was labeled by TRITC-conjugated phalloidin. Mitochondria were labeled by 200 nM MitoTracker^®^ Red CMXRos (Life Technologies) for 30 min at 20°C in live cells and followed by fixation. CT was labeled by primary antibody B2C generated in mouse (Furukawa et al., 1992) and FITC-conjugated anti-mouse IgG. Profilin I was labeled by antibody obtained as a gift from A. A. Noegel (University of Cologne, Germany) (Haugwitz et al., 1994), and FITC conjugated anti-mouse IgG. Fluorescent-conjugated antibodies and phalloidin were obtained from Sigma-Aldrich. Microscopy was performed using Applied Precision DeltaVision I microscope-imaging system (GE Healthcare Bio-Sciences, Pittsburgh, PA). Images were assembled using Photoshop CS6 (Adobe Systems, San Jose, CA). For experiments to determine model Hirano body sizes, images were recorded on a Zeiss IM35 microscope equipped with a CCD 300-T-RC camera (Dage MTI, Michigan City, IN) controlled by Scion Image software (Fredrick, MD).

### Drug treatment

CK666 (Sigma-Aldrich) was dissolved in DMSO to a stock concentration of 50 mM. Cells were incubated in HL-5 media for 6 h, 12 h and 18 h in the absence or presence of 100 µM CK666. For each sample with CK666, a solvent control incubated with equal concentration of DMSO for the same length of time was included.

### Determination of the size of model Hirano bodies and proportion of model Hirano body-forming cells

Microscopy images were taken in contiguous fields until a total of 200 model Hirano bodies were imaged. The total cell number in all the fields was also counted to calculate the proportion of cells that formed model Hirano bodies. The area of model Hirano bodies was measured using ImageJ (Schneider et al., 2012). Experiments were repeated three times in independent trials. The difference between the size distributions of model Hirano bodies in cells treated with CK666 and control cells were tested by exact test of goodness-of-fit. The difference between the proportions of cells that generated model Hirano bodies with or without the presence of CK666 was tested by Student's *t*-test. The difference between the size distributions of model Hirano bodies in [AS]proA and control cells was tested by Chi square test of goodness-of-fit.
